# Identifying Potential Hosts of Short-Branch Microsporidia

**DOI:** 10.1007/s00248-020-01657-9

**Published:** 2021-01-09

**Authors:** Annemie Doliwa, Micah Dunthorn, Erika Rassoshanska, Frédéric Mahé, David Bass, Camila Duarte Ritter

**Affiliations:** 1 Eukaryotic Microbiology, University of Duisburg-Essen, Universitätsstrasse 5, S05 R04 H83, 45141 Essen, Germany; 2 Centre for Water and Environmental Research (ZWU), University of Duisburg-Essen, 45141 Essen, Germany; 3 CIRAD, UMR BGPI, F-34398 Montpellier, France; 4 BGPI, Université de Montpellier, CIRAD, IRD, Montpellier SupAgro, Montpellier, France; 5 Centre for Environment, Aquaculture and Fisheries Science (Cefas), Barrack Road, Weymouth, Dorset, DT4 8UB UK; 6 Department of Life Sciences, The Natural History Museum, Cromwell Road, London, SW7 5BD UK; 7 Sustainable Aquaculture Futures, University of Exeter, Exeter, EX4 4QD UK

**Keywords:** Neotropics, Network analyses, Parasites, Protists, Soil biodiversity

## Abstract

Microsporidia are obligate parasites that are closely related to Fungi. While the widely known “long-branch” Microsporidia infect mostly metazoans, the hosts of “short-branch” Microsporidia are only partially characterized or not known at all. Here, we used network analyses from Neotropical rainforest soil metabarcoding data, to infer co-occurrences between environmental lineages of short-branch microsporidians and their potential hosts. We found significant co-occurrences with several taxa, especially with Apicomplexa, Cercozoa, and Fungi, as well as some Metazoa. Our results are the first step to identify potential hosts of the environmental lineages of short-branch microsporidians, which can be targeted in future molecular and microscopic studies.

**Supplementary Information:**

The online version contains supplementary material available at 10.1007/s00248-020-01657-9.

Environmental DNA sequencing studies have uncovered numerous protistan parasitic groups in different environments. For example, apicomplexans dominate soils in Neotropical rainforests [1] and Syndiniales are likewise species-rich in marine waters [2]. At least at larger taxonomic levels, it is relatively straightforward to infer the hosts of these protistan parasites: the apicomplexans mostly infect metazoans [3] and the Syndiniales infect metazoans and other protists [4]. However, we do not always know so clearly who are the hosts for other protistan parasite groups uncovered in environmental DNA sequencing studies. One such example of this lack of knowing who are the potential hosts are the “short-branch” microsporidians [5].

The short-branch microsporidians form a basal clade leading up to the more widely known “long-branch” Microsporidia [5]. Long-branch Microsporidia are mostly parasites of metazoans [6], but some can infect ciliates and other protists [7]. While the long-branch Microsporidia have highly reduced genomes and complex polar filaments that allow the penetration of cells, the short-branch microsporidians have less reduced genomes and they lack fully developed polar filaments [5]. The short-branch microsporidians include the partially characterized *Paramicrosporidium* that are parasites of *Saccamoeba limax* [8] and *Vannella* [9], *Mitosporidium* that are parasites of the crustacean *Daphnia* [10], as well as *Morellospora*, an amoeba parasite from the same clade as *Mitosporidium* [11], and *Nucleophaga*, a parasite of *Thecamoeba* [12]. The short-branch microsporidians also include numerous environmental lineages recently uncovered in a re-analysis of a metabarcoding study of Neotropical rainforest soils [5] and other environments [5, 13]. Presumably all of these environmental lineages phylogenetically assigned to the short-branch microsporidians are likewise parasitic; it is unknown, though, who are their potential microbial- or macro-organismic hosts, or where to even begin to look for them in environments as species-rich as tropical forests.

A novel approach to evaluating the diversity of protistan parasites and their hosts in metabarcoding datasets was recently demonstrated [14]. Using linear regression models, Singer et al. [14] showed that the abundances of apicomplexans and their metazoan hosts positively correlated across alpine sites in Switzerland. That type of analysis is dependent in part, though, on knowing what are the potential hosts through previous observations. Another approach to unravel potential host-parasite relationships when the hosts are unknown is to use co-occurrence network analyses. Although network analyses based on co-occurrences do not confirm biotic interactions [15], co-occurrence network can highlight potentially interesting taxonomic groups as potential hosts.

We used a co-occurrence network built from metabarcoding data from Mahé et al. [1]. Briefly, the data came from soils collected in lowland rainforests in Costa Rica, Panama, and Ecuador. The soils were amplified using broad eukaryotic primers for the V4 region of SSU-rRNA [16] and sequenced with Illumina MiSeq. After initial cleaning steps, reads were clustered into operational taxonomic units (OTUs) with swarm [17] and taxonomically assigned using the PR^2^ database [18]. Most of the OTUs were assigned to different protistan taxa, while others were assigned to Fungi and Metazoa. From this original data, refinements of the taxonomic assignments placed 974 OTUs into the short-branch microsporidia [5]. We calculated the richness of these OTUs with vegan v.2.5-6 [19], and compared the exclusive and shared OTUs by country with a Venn diagram [20] in R v.3.6.3 [21].

Representative sequences from all eukaryotic OTUs were used to construct a co-occurrence network with the NetworkNullHPC script (https://github.com/lentendu/NetworkNullHPC). In this network, the OTUs are represented as nodes, and a statistically significant Spearman correlation between two OTUs is represented by an edge between them. The network contains only OTUs with a significant co-occurrence with at least one other OTU, using a set of null models following Connor et al. [22]. The resulting co-occurrence matrix was analyzed in R with tidyverse v.1.3.0 [23] and igraph v.1.2.4.2 [24], then explored and visualized with Gephi v.0.9.2 [25] using the Yifan Hu layout. The network was filtered for short-branch microsporidians and their correlating nodes, then further explored with a Sankey diagram made in networkD3 v.0.4 [26]. Spearman correlation was used as a value to link short-branch microsporidia and their correlating nodes in the Sankey diagram as it is a ponderation between the number of edges and the strength of the correlation among nodes. Additionally, we ran networks analysis separatly for the 15 registered in our general network Microsporidia OTUs. For each of these Microposridia we just include plots that they are present (Fig. S1).

The co-occurrence network consisted of 14,329 edges involving 368 nodes (approximately 2.40% of all OTUs in the dataset). Costa Rica had the highest richness of short-branch Microsporidia, and the highest number of exclusive OTUs (Figs. S2 & S3). However, just 15 widespread microsporidian OTUs were present in the network, corresponding to approximately 1.54% of all their OTUs in the dataset (Fig. 1). Of these OTUs, 11 had a closest taxonomic assignment to the *Paramicrosporidium*, and four to the *Mitosporidium*, although the OTUs likely form independent environmental lineages (Table S1). Filtering the co-occurrences for correlations only associated with these 15 short-branch microsporidian OTUs resulted in 1223 edges involving 244 nodes, with 768 edges belonging to OTUs assigned to the “paramicrosporidium” and 455 edges to OTUs assigned to the “mitosporidium” (Fig. 2; Tables S2 and S3). Although all known hosts from the studied clades are either Metazoa or Amoebozoa [8–10], the three most prominent groups co-occurring with the short-branch microsporidians are the Cercozoa, Fungi, and Apicomplexa. The two largest groups in the cercozoans to form co-occurrences were the largely bacterivorous testate amoebae in the Thecofilosea and Euglyphida. Within the Fungi, the largest groups were the Chytridiomycota and the Ascomycota, that are mostly found in those tropical soils in yeast-forming stages [27]. Most of the apicomplexans were in the Gregarinasina, which are parasites of invertebrates and dominated the soil protistan communities in the tropical forests [1]. Some other groups that also co-occurred with the short-branch microsporidians included the already known hosts Amoebozoa and Metazoa, and also Endomyxa, Ciliophora, and Oomycota. The few metazoans in the networks were assigned to the Nematoda and Annelida (Table S4).

**Fig. 1:**
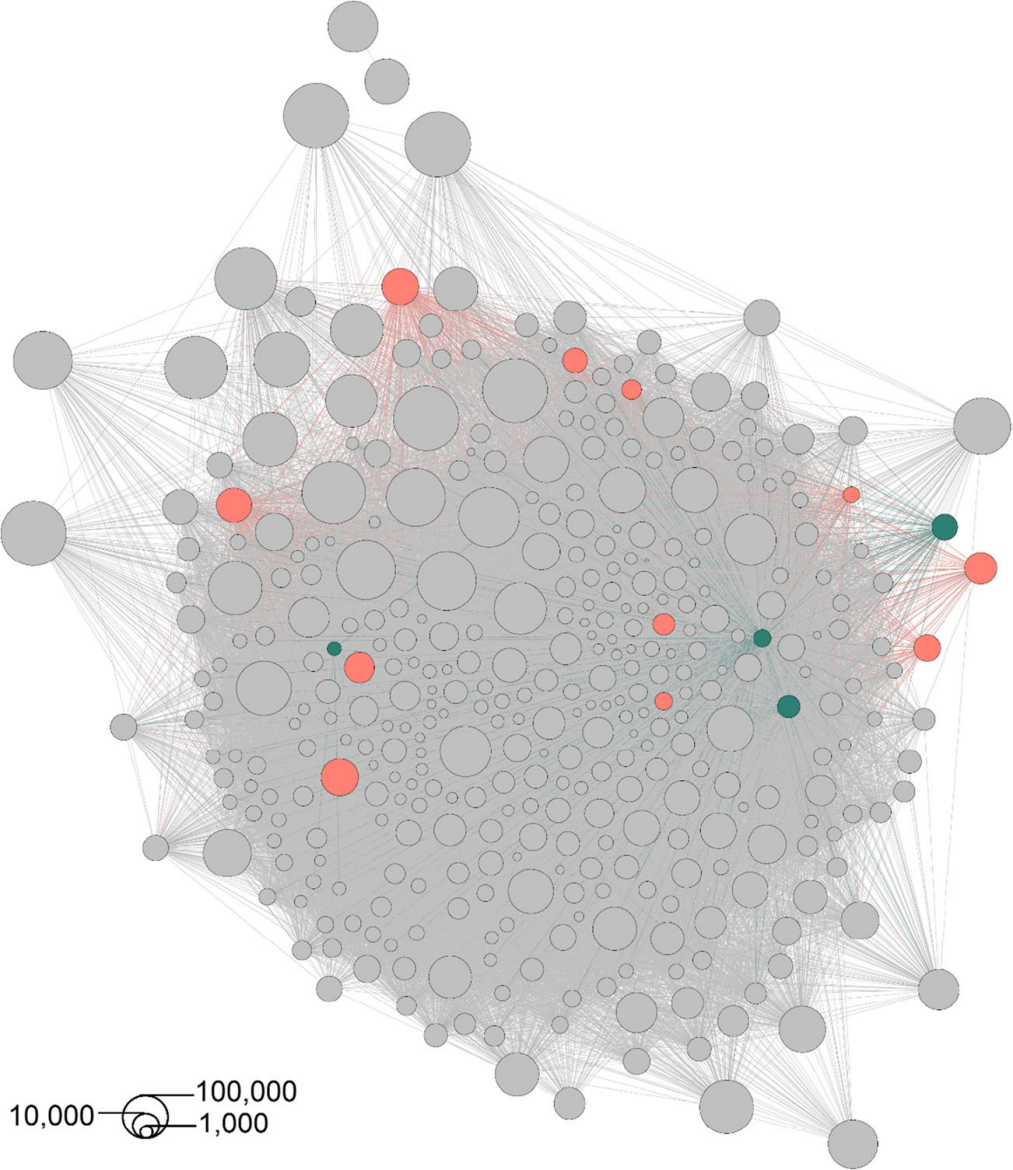
Co-occurrence network with OTUs as nodes and correlations as edges; the node size illustrates the abundance of the OTU. Mitosporidian OTUs are highlighted in turquois and paramicrosporidian OTUs in red

**Fig. 2:**
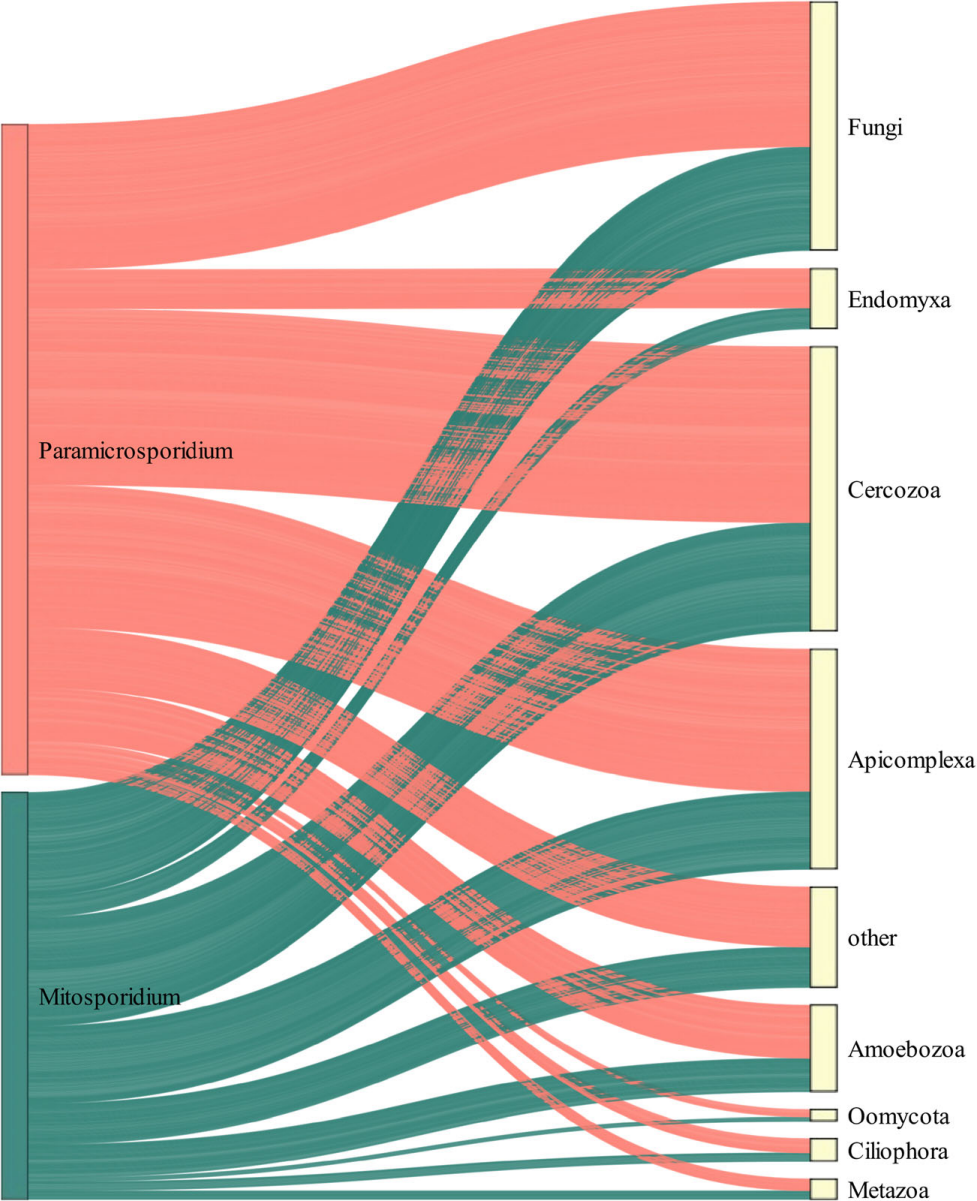
Sankey diagram showing the edges between microsporidian OTUs (left) and their target OTUs (right) in the co-occurrence network. Edges with paramicrosporidian OTUs are marked in red, those with mitosporidian OTUs are colored as turquoise

Although we found protists, fungi, and metazoans co-occurring with the short-branch microsporidians, the network analyses do not directly demonstrate that they are actual hosts. Co-occurrences can be inferred because of similar environment preferences, and actual biotic interactions may not have been inferred because the signal was too weak in the data [15]. Additionally, some of the co-occurrences here could have been inferred just because the cercozoans, fungi, and apicomplexans were extremely OTU-rich in the dataset. Potentially more of the short-branch microsporidians could have metazoan hosts, but the use of the SSU-rRNA environmental sequences likely underestimated their diversity, highlighted by the low proportion (1.54%) of short-branch Microsporidia OTUs registered in our network. Yet, we inferred co-occurrences between four annelids and one nematode, which are known to be involved in host-parasite associations with long-branch Microsporidia [e.g., 28, 29].

Even in light of these potential limitations, the co-occurrence networks here highlight taxa that should be evaluated further for being the hosts of the environmental linages of short-branch microsporidians in complex Neotropical rainforest communities. These additional observations could include fluorescence in situ hybridization (FISH) probes designed for the short-branch microsporidians and used on cell isolates of cercozoans, fungi, apicomplexans, and possibly metazoan. Furthermore, the co-occurrence network approach proposed here can be used to identify potential hosts of other parasiticprotists uncovered in environmental DNA sequencing studies of in different complex environments.

## Supplementary Information

ESM 1 (DOCX 1.62 mb)

## Data Availability

The data is available at Mahé et al. (2017).
