# Supplementary material for: Identifying Potential Hosts of Short-Branch Microsporidia
Source: Microb Ecol. 2021 Jan 9;82(2):549–53. doi: 10.1007/s00248-020-01657-9 (PMC8384821; doi:10.1007/s00248-020-01657-9)
Supplement: Supplementary file 1 — (DOCX 1.62 mb) [file 248_2020_1657_MOESM1_ESM.docx]

**Material Supplementary for:**

**Identifying Potential Hosts of Short-Branch Microsporidia**

Annemie Doliwa^1^, Micah Dunthorn^1,2^, Erika Rassoshanska^1^, Frédéric Mahé^3,4^, David Bass^5,6,7^, Camila Duarte Ritter^1^

^1^ Eukaryotic Microbiology, University of Duisburg-Essen, Universitätsstrasse 5, S05 R04 H83 D-45141 Essen, Germany

^2^ Centre for Water and Environmental Research (ZWU), University of Duisburg-Essen, D-45141 Essen, Germany

^3^ CIRAD, UMR BGPI, F-34398 Montpellier, France

^4^ Centre for Environment, Aquaculture and Fisheries Science (Cefas), Barrack Road, Weymouth, Dorset DT4 8UB, UK

^5^ Centre for Environment, Aquaculture and Fisheries Science (Cefas), Barrack Road, Weymouth, Dorset DT4 8UB, UK 6 Department of Life Sciences, The Natural History Museum, Cromwell Road, London SW7 5BD, UK

^7^ Sustainable Aquaculture Futures, University of Exeter, Exeter EX4 4QD, UK

*Corresponding author.

*Email address:* [camila.duarte-ritter@uni-due.de](mailto:camila.duarte-ritter@uni-due.de); kmicaduarte@gmail.com

Contents

[Table S1 3](#_Toc53662147)

[Table S2. 4](#_Toc53662148)

[Table S3. 5](#_Toc53662149)

[Table S4. 6](#_Toc53662150)

[Figure S1 7](#_Toc53662151)

[Figure S2. 8](#_Toc53662152)

[Figure S3. 9](#_Toc53662153)

[References 10](#_Toc53662154)

Table S1**.** Additional taxonomic information for the 15 SB-microsporidian OTUs in the analyzed co-occurrence network.

Table S2. Overview of the number of edges between the Mitosporidian or Paramicrosporidian and the different taxa, divided according to the individual SB-microsporidian OTUs. The letters a – f refer to the taxonomic information for the SB-microsporidian OTUs given in Table S1.

Table S3. Overview of the total number of edges per taxon.

Table S4. Further taxonomic information about the metazoan OTUs that showed a significant correlation with the SB-microsporidian OTUs.

**
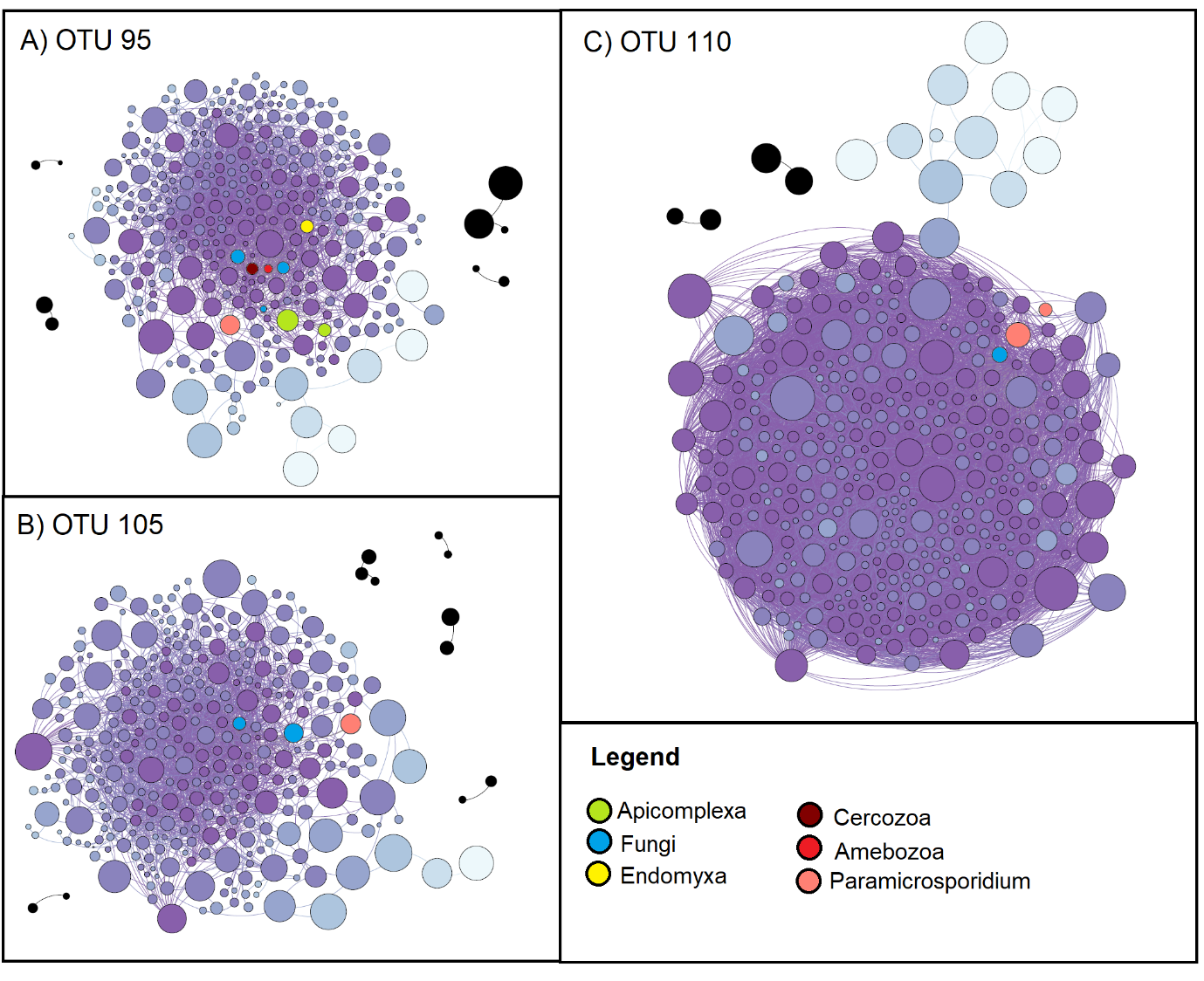
**

Figure S1**.** Co-occurrence networks with just plots where either the Paramicrosporidium A) OTU_95 or B) OTU_110 are presents. The OTUs are represented as nodes and correlations as edges; the node size illustrates the abundance of the OTU. The purple tons are related with more close association with the focal OTUs. Black Nodes are OTUs not connected in any level with the focal OTU. The others OTUs present in the main network analysis were not present when we restrict for just the plots the they are presents.

**
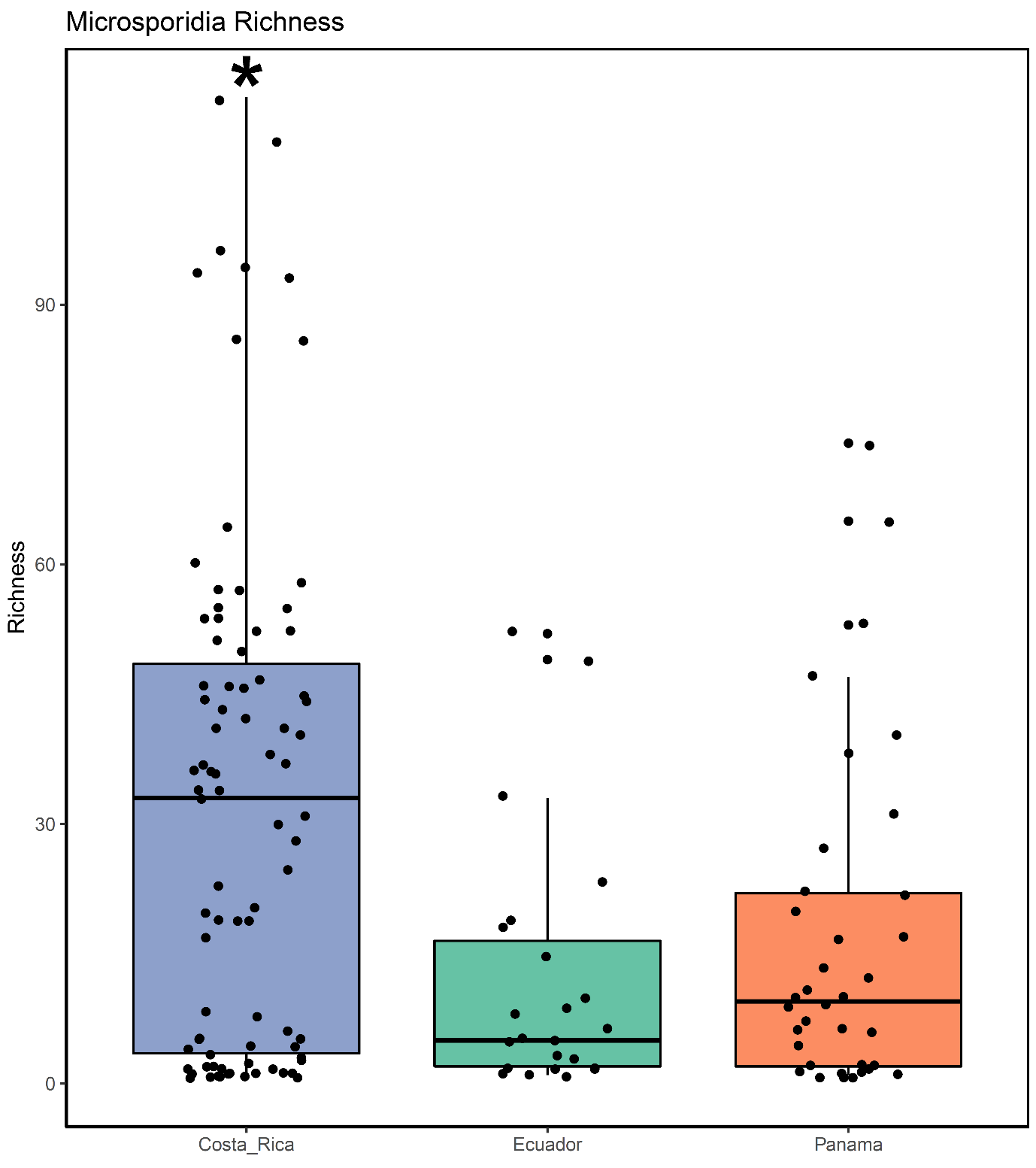
**

Figure S2. Richness of Microsporidia by country. Costa Rica has the highest and significant richness (Anova test: F value = 8.619. p = 0.0002). Turkey posthoc test to compare among countries showed significant difference between Costa Rica and Ecuador (Diff = 15.7, p = 0.005) and Costa Rica and Panama (Diff = -20.25, p = 0.002), but not between Ecuador and Panama (Diff = -4.5, p = 0.78). All analyses were calculated with vegan v.2.5-6 [1] in R v.3.6.3 [2].


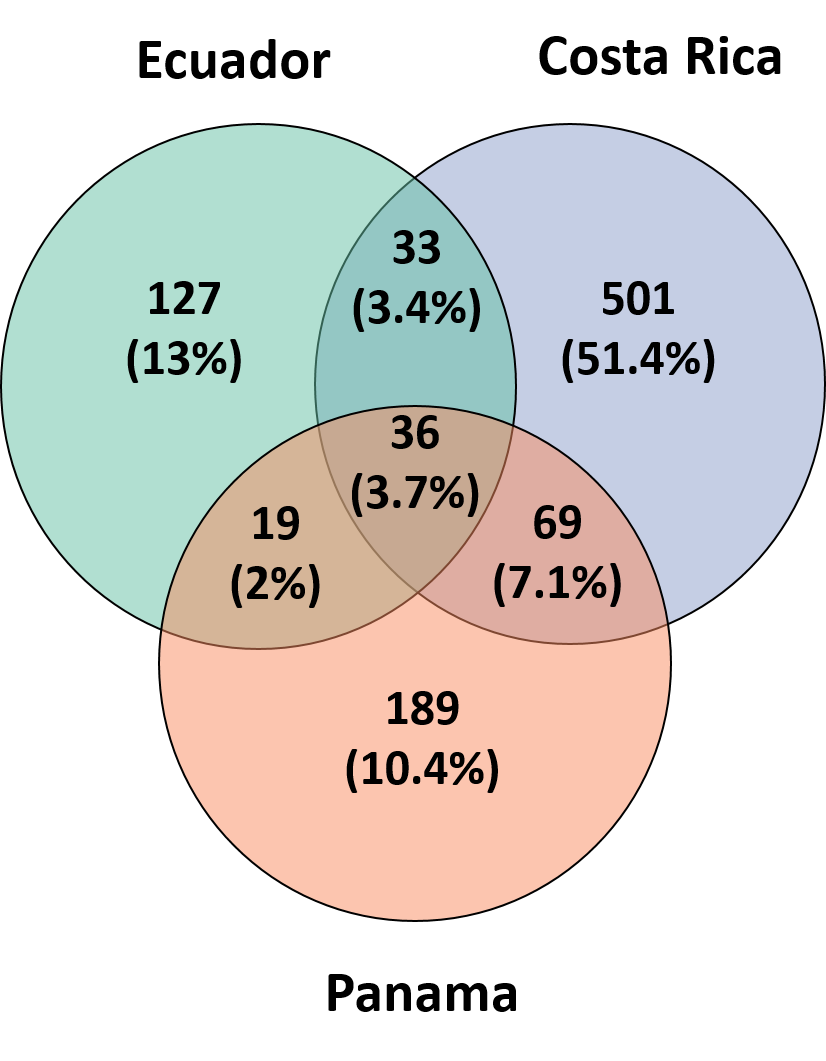


Figure S3. Number of Microsporidian OTUs exclusive and shared by country. Costa Rica has the highest number of exclusive OTUs. The number of shared Microsporidian OTUs between countries is very low. The Venn diagram was constructed using the online tool Venny 2.0 [3].

# References

1. Oksanen J, Blanchet FG, Kindt R, et al (2019) Vegan: Vegan: Community Ecology Package. R package Version 2.5-6. . http://cranr-project.org>. Accessed at 15 June of 2020.

2. R Core Team (2018). R: A language and environment for statistical computing. R Foundation for Statistical Computing, Vienna, Austria. URL https://www.R-project.org/. Accessed at 15 June of 2020.

3. Oliveros JC (2007) VENNY. An interactive tool for comparing lists with Venn Diagrams. http://bioinfogpcnbcsices/tools/venny/index.htm. Accessed at 21 July of 2020.
